# Somatoform and dissociative disorders in children and adolescents: A comparative study

**DOI:** 10.4103/0019-5545.46073

**Published:** 2005

**Authors:** Savita Malhotra, Gagandeep Singh, Ashwin Mohan

**Affiliations:** * Professor; ** Senior Lecturer; *** Consultant Psychiatrist

**Keywords:** Somatoform disorders, dissociative disorders, children, adolescents

## Abstract

**Background:**

Somatoform and dissociative (conversion) disorders in adults have been reported to have a close relationship because of a diagnostic overlap and comparable aetiological models. The literature on these disorders in children and adolescents is scarce.

**Aim:**

The present study attempted to compare these two disorders in children and adolescents since antecedents of these disorders are said to be laid in childhood.

**Methods:**

Case files of 118 patients (69 of somatoform disorders and 49 of dissociative disorders) were reviewed and the two groups were compared with respect to sociodemographic profile, clinical profile, neurotic traits, behavioural problems, temperament, intelligence and family dysfunction.

**Results:**

Age at presentation and intelligence were significantly higher in those with somatoform disorders than in those with dissociative disorders. Patients with dissociative disorders had a significantly higher number of co-morbid somatoform symptoms.

**Conclusion:**

Somatoform and dissociative disorders are closely linked.

## INTRODUCTION

Ever since the concept of ‘wandering womb’ was proposed by Hippocrates, hysteria, though ridden with controversies, has occupied an important place in our nosological system.^1^ With the introduction of modern classification systems (ICD-10 and DSM-IV), ‘hysteria’ is said to be represented mainly by two types of disorders—dissociative disorders and somatoform disorders.^2^

Typically, patients with dissociative disorders report symptoms that are produced as a result of a disturbance or alteration in the normally integrative function of identity, memory or consciousness. Patients report symptoms such as amnesia, fugue, depersonalization or altered personality. Somatoform disorders, on the other hand, are characterized by recurrent multiple somatic complaints which are not accounted for by any underlying physical illness.^2^

Within the two main classification systems (ICD-10 and DSM-IV), differences exist in terms of inclusion of disorders into one category or the other. With a purely aetiological approach, ICD-10 clubs patients with disorders characterized by psychological symptoms (dissociative amnesia, dissociative fugue, trance and possession disorder, Ganser syndrome, multiple personality disorder) along with disorders that have neurological manifestations (dissociative stupor, dissociative motor disorder, dissociative convulsions, and dissociative anaesthesia and sensory loss). It is presumed that in these disorders ‘dissociation’ serves as the basic mechanism and only the symptomatic presentation differs. In ICD-10, somatoform disorders include somatization disorder, undifferentiated somatoform disorder, persistent somatoform pain disorder, somatoform autonomic dysfunction, hypo-chondriacal disorder, somatoform disorders—others and somatoform disorder—unspecified.^3^

DSM-IV, on the other hand, adopts a phenomenological approach. Dissociative disorders here include only those that have ‘psychological’ symptomatic presentation. Disorders with a neurological symptomatic presentation are included under the category of ‘conversion disorders’ in the broad category of somatoform disorders.^4^

In recent years, there has been increasing research on ‘conversion disorders’ and ‘dissociative disorders’ as given in DSM-IV, with the aim of ascertaining a relationship between these two disorders. Both disorders have been found to have significant co-morbidity with one another (15%–30%).^2^ A significant number of patients with conversion disorder have been reported to have dissociative symptoms and vice versa.^5^ Both groups of patients have been reported to have a comparable element of associated anxiety, which is presumed to be the causative mechanism in both disorders.^1^ These findings have led some authors to comment that these two disorders are in fact closely related, thereby supporting the position held by ICD-10 of clubbing these together.

In recent years, further attempts have been made to find out the relationship between dissociative (conversion) and somatoform disorders. A close association between the two has been suggested since the early nineteenth century. In 1859, Briquet concluded that hysteria was the basis of diagnosis for somatoform disorders. Pierre Janet explained that in dissociation, memories of traumatic experiences might be expressed as somatic symptoms.^2^

Empirical research has further shown that 5%–42% of patients with somatoform disorders have been reported to have multiple personality disorder, and patients with multiple personality disorder have many somatic symptoms.^6^ High rates of dissociation have been reported in patients with chronic pelvic pain.^7^

Few studies have attempted to compare the aetiology of these disorders. Patients with both somatoform and dissociation disorders have been reported to have experienced high ‘stress’. In patients with somatoform disorders, the stress may be in the form of adverse life events, and disturbed interpersonal and family dynamics. In patients with dissociative disorders traumatic experiences, mainly sexual abuse, may be the stressors.^8^,^9^ Both disorders have been linked to a higher prevalence of various personality disorders (avoidant, self-defeating, passive-aggressive and dependent personality disorders).^10^,^11^

From the available literature, it is apparent that somatoform and dissociative disorders are comparable. It is therefore important to ascertain the relationship between these disorders to determine their nosological position and to understand their aetiology.

Research on these disorders has mainly concentrated on adults. However, somatoform and dissociative disorders are being increasingly recognized in children.^7^,^12^–^19^ It has also been demonstrated that the onset of somatoform and dissociative disorders in adulthood is linked to parental dysfunction and family psychopathology in childhood.

The present study compares the sociodemographic and clinical profile, neurotic traits and behavioural problems, intelligence and family dysfunction in children with somatoform and dissociative disorders. It was presumed that these variables would be comparable in both disorders, hence confirming their close relationship.

## METHODS

The sample of patients for the study was drawn from children attending the Child and Adolescent Psychiatry (CAP) Clinic of our institute between 1996 and 2000. This is a psychiatric unit in a general hospital setting to which patients may come directly or are referred to by other medical or surgical specialties. All patients are seen by two residents, one junior (MD trainee) and one senior (qualified psychiatrist with an MD degree), and discussed with a consultant for advice on diagnosis and management. Evaluation of patients is done as per a semistructured proforma and the diagnosis is based on ICD-10. Patients requiring inpatient treatment are admitted to the inpatient facility under the same consultant with a team of two residents, who are directly involved in the management of the patient.

All cases of somatoform and dissociative disorders registered during the period 1996–2000 formed the study sample. Case files of each patient were reviewed for details on sociodemographic and clinical variables. The presence of mental retardation and age at presentation more than 14 years served as exclusion criteria. The symptom profile was derived from common symptoms reported in the literature and from the ICD-10 diagnostic criteria.

The study sample comprised 118 patients. Statistical analysis was done using frequency, percentage, mean and standard deviation for descriptive purposes and the chi-square test for group comparison.

## RESULTS

Table 1 gives the sociodemographic profiles of the two groups of patients, which were similar.

**Table 1:** Sociodemographic profile of patients with somatoform disorder (SD) and dissociative disorder (DD)

Variables	SD (*n*=69)	DD (*n*=49)	Chi-square (df=1)
Sex			
Males	25 (21.2)	23 (19.5)	0.33
Females	44 (37.3)	26 (22.0)	
Education (grade)			
Nil	2 (1.7)	0 (0.0)	0.50
Second	4 (3.4)	3 (2.5)	
Third	1 (0.8)	3 (2.5)	
Fourth	2 (1.7)	2 (1.7)	
Fifth	40 (33.9)	31 (26.2)	
Eighth	20 (16.9)	10 (8.4)	
Religion			
Hindu	48 (40.7)	39 (33.0)	0.38
Sikh	20 (16.9)	10 (8.4)	
Others	1 (0.8)	0 (0.0)	
Income (Rs)			
0–2000	39 (33.0)	21 (17.8)	0.09
2001–5000	17 (14.4)	10 (8.4)	
5001 and above	13 (11.0)	18 (15.2)	
Locality			
Urban	50 (42.3)	35 (29.6)	1.0
Rural	19 (16.1)	14 (11.8)	

Df: degree of freedom

Chi-square value not significant.

Values in parentheses are percentages.

In 22 patients (19%), a precipitating factor could be elicited before the onset of the illness. On the other hand, in 36 patients (30.51%) a life event preceded the illness, with interpersonal problems in the family (9.32%), illness (7.62%), school difficulties (3.39%) and others (2.5%) being the commonest. Two patients (1.71%) reported examinations as a life event.

Twenty-three patients (19.5%) had a co-morbid disorder; 9 patients had a seizure disorder, 2 patients had moderate depressive episode, 2 had non-organic enuresis. One patient each in both the groups had mania without psychotic symptoms, recurrent depressive disorder, obsessive–compulsive disorder, conduct disorder and tic disorder. A co-morbid diagnosis of somatoform and dissociative disorder was made in only 3 patients.

The patients had a mean of 2.43 symptoms (±1.59); range 1–8. Patients with dissociative disorder (*n*=49) had a mean of 1.23 symptoms (±0.62); range 1–4, while patients with somatoform disorder (*n*=69) suffered from a mean of 2.81 symptoms (±1.61; range 1–7). Twenty-eight patients with a diagnosis of dissociative disorder had 2.07 somatoform symptoms (± 1.35; range 1–5). Eight patients with a diagnosis of somatoform disorder had dissociative symptoms with a mean of 1.0 symptom per patient.

As shown in Table 2, patients with somatoform disorder (*n*=69) were older as compared to patients with dissociative disorder (*n*=49) (p=0.047). The mean intelligence quotient of somatoform disorder patients (*n*=44, intelligent quotient was not determined in the others) was significantly higher (p=0.017) than in patients with dissociative disorder (*n*=34, intelligent quotient was not determined in the others). The mean duration of illness for the groups was 15.50 months (±20.70) with no intergroup differences. The mean number of total symptoms in the patients with somatoform disorders was significantly higher as compared to the dissociative disorders group (p<0.005).

**Table 2:** Comparison of sociodemographic and clinical variables in patients with somatoform disorder (SD) and dissociative disorder (DD)

Variables	SD (*n*=69) (mean)	DD (*n*=49) (mean)	*t*
Age	12.36±1.72	11.67±1.90	0.04*
Education (in years)	5.49±1.93	5.26±1.63	0.50
Duration of illness (in months)	13.29±20.94	18.61±20.16	0.17
Total symptoms	2.95±1.67	2.05±1.44	0.002**
Intelligence quotient	96.20±11.28 (*n*=44)	90.06±10.03 (*n*=34)	0.017*
Number of neurotic traits	0.79±1.18	1.02±1.26	0.33
Number of behavioural problems	0.26±0.65	0.32±0.74	0.61

* p<0.05;

** p<0.005

The two groups did not differ on the number of neurotic traits and behavioural problems.

The two groups did not differ in the variables of presence of impairment, secondary gain and family dysfunction (Table 3).

**Table 3:** Comparison of impairments, secondary gains and family dysfunction in patients with somatoform disorder (SD) and dissociative disorder (DD)

Variables	SD (*n*=69)	DD (*n*=49)	Chi-square (df=1)
Impairments			
Absent	30 (25.42)	30 (25.42)	0.060
Present	39 (33.05)	19 (16.10)	
Secondary gains			
Absent	27 (22.8)	21 (17.7)	0.65
Present	42 (35.5)	28 (23.7)	
Family dysfunction			
Absent	29 (24.5)	20 (16.9)	0.89
Present	40 (33.8)	29 (24.5)	

df: degree of freedom

Chi-square value not significant.

Values in parentheses are percentages.

Table 4 shows the temperamental characteristics of the patients, which were similar in both the groups.

**Table 4:** Temperamental characteristics of patients with somatoform disorder (SD) and dissociative disorder (DD)

Variables	SD (*n*=69)	DD (*n*=49)	Chi-square (df=1)
Activity			
Hyperactivity	2 (1.7)	5 (4.2)	0.24
Normal	66 (55.9)	43 (36.4)	
Low activity	1 (0.8)	1 (0.8)	
Rhythmicity			
Normal	62 (52.5)	46 (58.9)	0.66
Abnormal	7 (5.9)	3 (2.5)	
Approach–withdrawal			
Normal	62 (52.3)	46 (58.9)	0.66
Disturbed	7 (5.9)	3 (2.5)	
Adaptability			
Normal	60 (50.8)	44 (37.28)	0.63
Poor	9 (7.6)	5 (4.2)	
Mood			
Low	5 (4.2)	7 (5.9)	0.36
Normal	59 (91.5)	37 (31.3)	
Irritable	5 (4.2)	5 (4.3)	
Intensity of reaction			
Low	9 (7.6)	4 (3.4)	0.70
Normal	50 (42.3)	38 (32.3)	
High	10 (8.4)	7 (5.9)	
Threshold of responsiveness			
Low	12 (10.1)	10 (8.4)	0.9
Normal	53 (45.0)	36 (30.5)	
High	4 (3.4)	3 (2.5)	
Attention span			
Normal	57 (48.3)	49 (33.0)	0.86
Abnormal	12 (10.1)	10 (8.4)	
Persistence			
Normal	55 (46.6)	42 (35.6)	0.55
Abnormal	14 (11.8)	7 (0.5)	
Distractability			
Low	13 (11.0)	8 (6.7)	0.55
Normal	52 (44.0)	40 (3.9)	
High	4 (3.4)	1 (0.84)	

df: degree of freedom

Chi-square value not significant.

Values in parentheses are percentages.

Table 5 shows the distribution pattern of parental functioning in the patient population. The majority of parents were permissive, consistent, liberal, approving the interest of the child, protective, tolerant of deviances and did not have any undue expectations from the patients.

**Table 5:** Comparison of pattern of parental functioning in patients with somatoform disorder (SD) and dissociative disorders (DD)

Variables	SD (*n*=69)	DD (*n*=49)	Chi-square (df=1)
Permissiveness			
Permissive	57 (48.3)	36 (30.5)	0.33
Rigid	12 (10.17)	13 (11.0)	
Consistency			
Consistent	45 (38.1)	35 (29.6)	0.61
Inconsistent	24 (20.3)	14 (11.8)	
Liberal			
Strict	26 (22.0)	13 (11.0)	0.28
Liberal	43 (36.4)	36 (30.5)	
Approval of interest			
Approval	56 (47.4)	42 (35.6)	0.69
Disapproval	13 (11.0)	7 (5.9)	
Protection			
Protective	52 (44.0)	38 (32.2)	0.95
Non-protective	17 (14.4)	11 (9.3)	
Tolerance of deviation			
Tolerant	51 (43.2)	36 (30.5)	1.00
Non-tolerant	18 (15.2)	13 (11.0)	
Pressure/expectation			
Absent	49 (41.2)	35 (29.6)	1.00
Present	20 (16.9)	14 (11.6)	

df: degree of freedom

Chi-square value not significant.

Values in parentheses are percentages.

## DISCUSSION

The nosological status of disorders is decided according to diagnostic validation, aetiological mechanisms, and course and outcome. Studies attempting to examine relationship between somatoform and dissociative disorders point to a close relationship between these two disorders in terms of diagnostic overlap and some similarities in aetiological mechanisms (stress experienced and personality variables).

Somatoform and dissociative disorders in children are a largely underresearched area with a paucity of literature on the crucial areas of prevalence, aetiology, treatment, course and outcome.^7^

An attempt has been made in recent years to compare these two disorders and find out the relationship between them. It would be pertinent to attempt to examine their relationship in children since the distinction between these two disorders still needs to be established in children. It is also proposed that the foundation of these disorders is laid in childhood due to childhood abuse, neglect and parental dysfunction.

In this study, the sociodemographic profile of the two groups of patients was comparable, with patients in the somatoform disorder group being significantly older than those in the dissociative disorder group. No study has compared the age at onset of these two groups of disorders as was done in the present study. The results point to age being a variable in the determination of symptomatic presentation.

Positive evidence of the importance of the psychological factors, especially in patients with pain, have included the onset of pain symptoms after trauma/stress; disability or handicap out of proportion to the pain reported, clear secondary gains and exacerbation of symptoms linked to stressful events.^7^ In dissociative (conversion) disorder, symptoms are reported to occur after a particular stressful experience.^9^ Comparable impairments and secondary gains point to the importance of psychological factors and their similar role in the determination of symptoms in both the disorders.

An assessment of neurotic traits across the two disorders did not reveal any differences. Neurotic traits are reported to be antecedents of internalizing disorders.^21^ Since neurotic traits as such lack any discriminant validity, this lack of difference is expected.

Behavioural problems (stealing, lying, disobedience, truancy, etc.) are regarded as a part of externalizing disorders. The lack of difference between the two groups provides additional evidence for similarity in yet another parameter.

In adults, personality disorders (self-defeating, dependent, passive–aggressive, etc.) are reported to be common in patients with somatoform and dissociative disorders. In the present study the temperaments of children were assessed. No significant abnormality of temperament in the group as a whole was found. More comparative studies of temperamental differences need to be carried out.

Family dysfunction (as measured by family functioning, child–parent interaction and special family environmental condition) was found to be high but comparable in both the groups. Earlier studies on both these two disorders have reported significant family pathology, but no study has actually compared these variables in children.

The finding of a significantly higher intelligence quotient in somatoform disorder compared with dissociative disorder needs interpretation. The earlier concept of hysteria (which is now included in dissociative disorders) was understood as a form of emotional and cognitive immaturity with a lower intellectual repertoire. Our finding apparently confirms this psychodynamic observation.

Thirty-six of the 118 patients, i.e. 30.50% had both dissociative and somatoform symptoms with the mean total number of symptoms (in total patients) being 2.43. Twenty-eight patients with dissociative disorder had somatoform symptoms and 8 somatoform disorder patients had dissociative symptoms. Only 3 patients had co-morbid somatoform and dissociative disorder, which suggests that there is considerable overlap in the clinical profile.

The study had some important limitations in terms of its retrospective nature and lack of structured assessment. However, the strength of the study lies in the fact that it was done in children, in a non-western setting and the sample size was relatively large. Hence, its implications are important.

Somatoform disorders are a heterogeneous group of conditions, which historically have a separate origin. The only common factor is somatic symptoms in the absence of physical disease. Before the 1980s some patients with somatoform disorder and all patients with dissociative disorder would have been clubbed under the general rubric of ‘hysteria’. Though these disorders have now been classified separately, the validity of this is questionable. Till date, no study has tried to compare the two disorders on presumed aetiological variables. In the present study somatoform and dissociative disorders were found to be comparable. The study hence supports the earlier position held by Saxe *et al*.^2^ that dissociative and somatoform disorder are closely linked.
